# Persistently elevated osteopontin serum levels predict mortality in critically ill patients

**DOI:** 10.1186/s13054-015-0988-4

**Published:** 2015-06-26

**Authors:** Christoph Roderburg, Fabian Benz, David Vargas Cardenas, Matthias Lutz, Hans-Joerg Hippe, Tom Luedde, Christian Trautwein, Norbert Frey, Alexander Koch, Frank Tacke, Mark Luedde

**Affiliations:** Department of Medicine III, University Hospital RWTH Aachen, Pauwelsstrasse 30, 52074 Aachen, Germany; Department of Internal Medicine III, University of Kiel, Schittenhelmstrasse 12, 24105 Kiel, Germany

## Abstract

**Introduction:**

Inflammatory, autoimmune and metabolic disorders have been associated with alterations in osteopontin (OPN) serum levels. Furthermore, elevated serum levels of OPN were reported from a small cohort of patients with sepsis. We therefore analyzed OPN serum concentrations in a large cohort of critically ill medical patients.

**Methods:**

A total of 159 patients (114 with sepsis, 45 without sepsis) were studied prospectively upon admission to the medical intensive care unit (ICU) as well as after 3 days of ICU treatment and compared to 50 healthy controls. Clinical data, various laboratory parameters as well as investigational inflammatory cytokine profiles were assessed. Patients were followed for approximately 1 year.

**Results:**

We found significantly elevated serum levels of OPN at admission to the ICU and after 3 days of treatment in critically ill patients compared to healthy controls. OPN concentrations were related to disease severity and significantly correlated with established prognosis scores and classical as well as experimental markers of inflammation and multi-organ failure. In the total cohort, OPN levels decreased from admission to day 3 of ICU treatment. However, persistently elevated OPN levels at day 3 of ICU treatment were a strong independent predictor for an unfavorable prognosis, with similar or better diagnostic accuracy than routinely used markers of organ failure or prognostic scoring systems such as SAPS2 or APACHE II score.

**Conclusions:**

Persistently elevated OPN serum concentrations are associated with an unfavourable outcome in patients with critical illness, independent of the presence of sepsis. Besides a possible pathogenic role of OPN in critical illness, our study indicates a potential value for OPN as a prognostic biomarker in critically ill patients during the early course of ICU treatment.

**Electronic supplementary material:**

The online version of this article (doi:10.1186/s13054-015-0988-4) contains supplementary material, which is available to authorized users.

## Introduction

Sepsis and septic shock represent major causes of mortality in patients referred to intensive care units [1]. Sepsis is defined by a Systemic Inflammatory Response Syndrome (SIRS) in the context of infection [2]. This response is characterized by both pro-inflammatory and anti-inflammatory phases and involves the expression and secretion of distinct pro- and anti-inflammatory mediators such as cytokines and chemokines by different immune and parenchymal cells [3]. Despite the rapid progresses of the “omics” research resulting in such sepsis-related panels of chemokines and cytokines, there is a high demand for new biomarkers that can help to better risk stratify patients and assist clinical decision-making in allocating resources of intensive care treatment [4].

Osteopontin (OPN) represents a phosphorylated acidic glycoprotein that is involved in a broad variety of physiological and pathological processes such as cancer, fibrosis, inflammation and heart disease [5–7]. Regarding inflammatory processes, OPN acts as a chemotactic factor for T cells, macrophages or neutrophils and modulates the function and differentiation of these inflammatory cells [8]. Moreover, mediators of sepsis and inflammation, including tumour necrosis factor (TNF) and interleukin (IL)-1, stimulate the expression of OPN on a transcriptional level, which appears critical for the recruitment and activation of macrophages in inflammation and infection [9]. Consequently, elevated serum and tissue levels of OPN were found in different diseases associated with systemic or focal inflammation, such as tuberculosis [10], multiple sclerosis [11], lupus erythematosus [12] and Crohn’s disease [13], thus suggesting that circulating OPN may hold potential as a biomarker for inflammatory and infectious diseases. Recently, OPN has been introduced as a novel biomarker in cardiac diseases, predicting the prevalence and prognosis of chronic and acute congestive heart failure and pulmonary hypertension [14–17].

Despite the emerging roles of OPN in the regulation of inflammation and immunity, its functional involvement in systemic infections remains to be elucidated. Moreover, the diagnostic and prognostic value of OPN measurements in critically ill patients is currently unclear [18]. We therefore conducted a large study with critically ill patients at a medical intensive care unit (ICU) and performed longitudinal measurements of OPN serum concentrations during the first days of ICU treatment. The aim of this study was to address the regulation and diagnostic value of OPN serum concentrations in critical illness, sepsis and/or multi-organ failure. Finally, we assessed whether OPN serum levels can serve as a prognostic predictor for ICU and long-term survival.

## Methods

### Study design and patient characteristics

A total of 159 consecutive patients (99 male, 60 female; median age 66 years, range 20–90 years) that were admitted to the general Internal Medicine ICU at the University Hospital RWTH Aachen were enrolled into this study (Table 1). Patients, who were expected to have a short-term (<72 h) intensive care treatment due to post-interventional observation or acute intoxication, were not included into this study, following previously published practice [19, 20]. The medium length of stay at the ICU was 9 days (range 1–70 days). Patient data, clinical information and blood samples were collected prospectively. The clinical course of patients was observed in a follow-up period by directly contacting the patients, the patients’ relatives or their primary care physician. Patients that met the criteria proposed by the American College of Chest Physicians and the Society of Critical Care Medicine Consensus Conference Committee for sepsis, severe sepsis and septic shock were categorized as sepsis patients, the others as non-sepsis patients [21, 22]. Patients displaying a body mass index (BMI) of >30 kg/m^2^ at ICU admission (prior to any treatment) were defined as obese. Patients with a medical history of type 2 diabetes and a concomitant intake of diabetes-related medication were defined as diabetics. As a control population, we analyzed 50 healthy blood donors with normal values for blood counts, C-reactive protein (CRP) and liver enzymes.

**Table 1:** Baseline patient characteristics

Parameter	All patients	Non-sepsis	Sepsis
Number	159	45	114
Sex (male/female)	99/60	28/17	71/43
Age median (range) (years)	66 (20–90)	63 (20–85)	67 (21–90)
APACHE II score median (range)	18.5 (2–40)	16.5 (2–31)	19 (3–40)
SAPS2 score median (range)	44 (9–80)	50 (30–80)	44 (9–65)
ICU days median (range)	9 (1–70)	7 (1–44)	11 (1–70)
Death during ICU (%)	25.8	20.0	28.1
Death during ICU or follow-up (%)	46.5	42.2	48.2
Ventilation yes (%)	74.2	73.3	74.6
Body mass index (BMI)	25.4 (15.9–59.5)	25.2 (15.9–40.5)	25.6 (16.4–59.5)
Creatinine median (range) (mg/dl)	1.4 (0.2–21.6)	1 (0.2–7.9)	1.6 (0.2–21.6)
Albumin median (range) (g/l)	27 (6–44)	29.5 (18.7–44)	25.2 (6–41)
WBC median (range) (x10^3^/μl)	12.5 (0.1–208)	12.5 (1.8–27.3)	12.8 (0.1–208)
CRP median (range) (mg/dl)	140 (5–230)	17 (5–230)	186 (5–230)
Procalcitonin median (range) (μg/l)	1.2 (0.1–182.6)	0.2 (0.1–36.5)	3.1 (0.1–182.6)
Interleukin 6 median (range) (pg/ml)	97 (2–26000)	22 (4.5–250)	135 (2–26000)
Tumour necrosis factor median (pg/ml)	26 (4.1–4330)	14 (4.1–35)	35 (9.6–430)
INR	1.17 (0.94–4.64)	1.14 (0.95–3.45)	1.18 (0.94–4.64)
Osteopontin at admission median (ng/ml)	3358.5 (25.2–5884.4)	3166.9 (36.3–5782.6)	3504.8 (25.2–5884.4)
Osteopontin at day 3 median (ng/ml)	1981.8 (13.9–3613.9)	1997.6 (361.7–3577.0)	1948.7 (13.9–3613.9)

*APACHE* Acute Physiology and Chronic Health Evaluation, *CRP* C-reactive protein, *ICU* intensive care unit, *INR* international normalized ratio, *SAPS* Simplified Acute Physiology Score, *WBC* white blood cell count

The study protocol was approved by the local ethics committee and conducted in accordance with the ethical standards laid down in the Declaration of Helsinki (ethics committee of the University Hospital Aachen, RWTH University, Aachen, Germany, reference number EK 150/06). Written informed consent was obtained from the patient, his or her spouse or the appointed legal guardian.

### Determination and definitions of relevant parameters in critically ill patients

Serum was obtained at admission to the ICU before therapeutic intervention. For 65 patients, follow-up measurements were available at day 3 and day 7 of ICU treatment. All samples were immediately placed on ice, centrifuged, and the serum samples were stored at −80 °C. Interleukin 6 (IL-6), IL-10, TNF, procalcitonin (PCT), soluble urokinase plasminogen activator receptor (suPAR), NTproCNP, ghrelin, hyaluronic acid, a proliferation-inducing ligand (APRIL) and micro ribonucleic acid (miRNA)-133a were measured as described previously [23–29]. Glomerular filtration rates (GFR) were calculated on basis of serum cystatin C levels. ICU mortality was defined as death on ICU; overall mortality included death at the ICU or during the observation period (after discharge from ICU and hospital) [23–29].

### Determination of osteopontin serum concentrations by ELISA

Osteopontin (OPN) serum concentrations were analysed using a commercial enzyme immunoassay according to the manufacturer’s instructions (Immuno-Biological Laboratories, Minneapolis, MN, USA, Code No. 27158).

### Statistical analysis

All statistical analyses were performed with SPSS (SPSS Inc., Chicago, IL, USA) as previously described [25, 30]. In the tables, data are displayed as median and range considering the skewed distribution of most parameters. Differences between two groups were assessed by Mann–Whitney *U* test, and multiple comparisons between more than two groups have been conducted by Kruskal-Wallis ANOVA and Mann–Whitney *U* test for post hoc analysis. Box plot display a statistical summary of the median, quartiles and extreme values. The whiskers extend from the minimum to the maximum value excluding outside and far out values, which are displayed as separate points. An outside value (indicated by an open circle) was defined as a value that is smaller than the lower quartile minus 1.5 times the interquartile range, or larger than the upper quartile plus 1.5 times the interquartile range. A far out value was defined as a value that is smaller than the lower quartile minus three times the interquartile range, or larger than the upper quartile plus three times the interquartile range. All values, including “outliers”, have been included for statistical analyses. Correlations between variables have been analysed using the Spearman correlation tests, where values of *p* <0.05 were considered statistically significant. The prognostic value of the variables was tested by univariate and multivariate analysis in the Cox regression model. Kaplan-Meier curves and log-rank test calculations were performed to display the impact on survival. Receiver operating characteristic (ROC) curve analysis and the derived area under the curve (AUC) statistic provide a global and standardized appreciation of the accuracy of a marker or a composite score for predicting an event. ROC curves were generated by plotting sensitivity against 1-specificity. Optimal cutoff points were calculated with the highest Youden Index, also positive likelihood ratios (LHR+), negative likelihood ratios (LHR-) and diagnostic odds ratios for this cutoffs, as previously described by Ray et al. [31].

## Results

### Osteopontin serum concentrations are elevated in critically ill patients

**Fig. 1:**
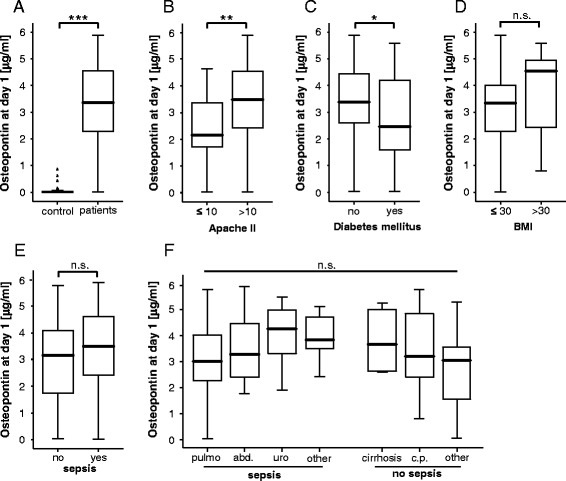
Serum osteopontin concentrations of critically ill patients at ICU admission. **a** Serum OPN concentrations at admission to the medical ICU were determined by ELISA and revealed significantly (*p* <0.001, ***U*** test) higher OPN levels in critically ill patients as compared with healthy controls. **b** Serum OPN levels at admission to the medical ICU are significantly (*p* = 0.005, *U* test) elevated in critically ill patients with high initial Acute Physiology and Chronic Health Evaluation (APACHE) II scores (>10) compared to patients with low APACHE II scores (≤10). **c** Serum OPN concentrations at admission to the ICU are significantly lower in patients with type 2 diabetes. **d** Serum OPN concentrations at admission to the medical ICU are independent of the presence of obesity in critically ill patients. **e** Patients that fulfilled sepsis criteria displayed no further increases in OPN serum levels at admission to the medical ICU. **f** OPN serum concentrations did not differ with regard to the disease aetiology in critically ill patients. pulmo, pulmonal; abd, abdominal; uro, urological; cirrh, cirrhosis; c.p., cardiopulmonary. Box plot are displayed, where the *bold line* indicates the median per group, the *box* represents 50 % of the values, and *horizontal lines* show minimum and maximum values of the calculated non-outlier values; *asterisks* and *open circles* indicate outlier values. ^*^ *p* <0.05, ^**^ *p* <0.01, ^***^ *p* <0.001. *ELISA* enzyme-linked immunosorbent assay, *ICU* intensive care unit, *OPN* osteopontin

Serum samples of critically ill patients were analysed at admission (that is, before specific therapeutic interventions) to the ICU and after 3 days of treatment. As shown in Fig. 1a, patients had significantly higher OPN serum concentrations at ICU admission as compared to healthy controls. Of note, high serum levels of OPN were associated with the severity of disease, since patients with high Acute Physiology and Chronic Health Evaluation (APACHE) II scores (>10) displayed a further increase in circulating OPN levels (median 3497.41 ng/ml, range 25.19–5884.44 ng/ml) compared to patients with low APACHE II scores (median 2157.59 ng/ml, range 36.30–4634.44 ng/ml; *p* = 0.005; Fig. 1b).

Serum levels of OPN can be influenced by metabolic disorders such as obesity and diabetes [32]. As these comorbidities might represent important confounding factors in medical patients admitted to the ICU, we performed subgroup analyses in our cohort. Serum OPN levels were even slightly reduced in patients with pre-existing type 2 diabetes upon admission to the ICU and did not vary dependent on the presence of obesity (Fig. 1c, d). In addition, no correlations were found between OPN concentrations and patients’ age or sex (not shown). These data indicate that elevated OPN levels indeed primarily relate to disease severity in critically ill patients.

Based on a recent pilot study suggesting that OPN might represent a novel biomarker for predicting sepsis in critically ill patients [18], we next tested whether serum OPN differed between ICU patients with or without sepsis. Among the 159 critically ill patients enrolled into this study, 114 patients fulfilled the criteria of sepsis (Table 2) [21, 22]. Within the sepsis cohort, pneumonia represented the predominant aetiology of critical illness (n = 62), while most patients with non-septic diseases suffered from cardiovascular diseases (n = 14) followed by decompensated liver cirrhosis (n = 10). Other aetiologies for non-septic disease included gastrointestinal (n = 6) and non-gastrointestinal (n = 5) bleedings, intoxications (n = 2), acute pancreatitis (n = 4) and diabetic ketoacidosis (n = 4). In contrast to the previous study [18], we found only a moderate increase in OPN serum concentrations at admission in patients with sepsis compared to patients without septic disease (Fig. 1e). Moreover, when the impact of the underlying aetiology was analysed no significant differences became apparent between the different subgroups both in septic as well as in non-septic patients (Fig. 1f).

**Table 2:** Disease aetiology of the study population

	Sepsis	Non-sepsis
	114 (71.7)	45 (28.3)
Aetiology of sepsis critical illness		
Site of infection n (%)		
Pulmonary	62 (39.0)	
Abdominal	20 (12.6)	
Urogenital	8 (5.0)	
Other	24 (15.1)	
Aetiology of non-sepsis critical illness		
n (%)		
Cardiopulmonary disease		14 (8.8)
Decompensated liver cirrhosis		10 (6.3)
Non-sepsis other		21 (13.2)

### Kinetics of osteopontin serum concentrations during early ICU treatment

**Fig. 2:**
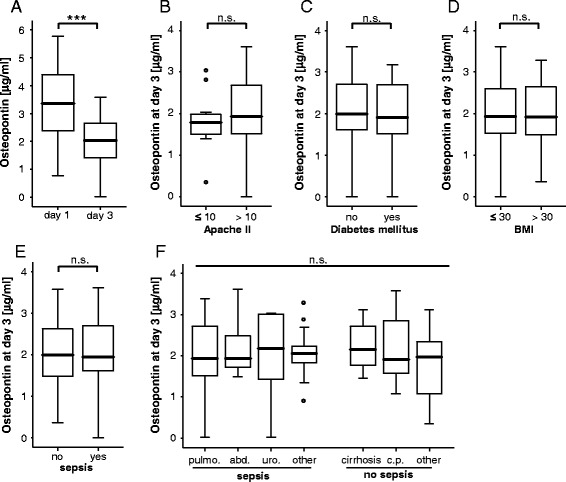
Serum osteopontin concentrations of critically ill patients after 3 days of ICU treatment. **a** Serum OPN concentrations after 3 days of ICU treatment were analysed by ELISA. In the total patient cohort, OPN levels were significantly lower when compared to the values at admission to the ICU (*p* <0.001, *U* test). **b** Serum OPN levels after 3 days of ICU treatment were not further elevated in patients with a more severe diseases according to the initial Acute Physiology and Chronic Health Evaluation (APACHE) II scores (>10) compared to patients with low APACHE II scores (≤10). **c** Serum OPN concentrations at day 3 of ICU treatment are independent on the presence of type 2 diabetes. **d** Serum OPN concentrations at day 3 of ICU treatment are independent on the presence of obesity. **e** Patients that fulfilled sepsis criteria displayed no further increases in OPN serum levels at day 3 of ICU treatment compared to patients with non-septic disease. **f** OPN serum concentrations did not differ with regard to the disease aetiology in critically ill patients. pulmo, pulmonal; abd, abdominal; uro, urological; cirrh, cirrhosis; c.p., cardiopulmonary. Box plot are displayed, where the *bold line* indicates the median per group, the *box* represents 50 % of the values, and *horizontal lines* show minimum and maximum values of the calculated non-outlier values; *asterisks* and *open circles* indicate outlier values. N values are given in the figures and tables. ^***^ *p* <0.001. *ELISA* enzyme-linked immunosorbent assay, *ICU* intensive care unit, *OPN* osteopontin

We next analysed the kinetics of OPN serum concentrations during the first 3 days of ICU treatment. Interestingly, despite remaining elevated compared to healthy controls, longitudinal OPN measurements revealed overall a strong and significant decrease between admission and day 3 (Fig. 2a). Of note, this was observed for the total cohort of all critically ill patients as well as for the subgroup of sepsis and non-sepsis patients (not shown).

We next compared OPN serum levels at day 3 of ICU treatment between the different predefined subgroups of ICU patients, namely low vs. high APACHE II scores (Fig. 2b), pre-existing diabetes (Fig. 2c), obesity (Fig. 2d), sepsis (Fig. 2e), or different disease aetiologies (Fig. 2f). None of these subgroup analyses revealed significantly regulated OPN serum levels at day 3.

### Osteopontin levels at admission to the ICU and after 3 days of ICU treatment are correlated to markers of organ function, inflammation and prognosis scores

**Fig. 3:**
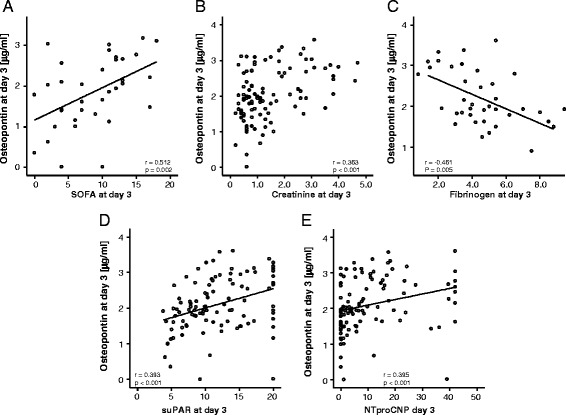
Association of osteopontin serum concentrations with serum levels of experimental markers of critical illness. **a-e** Serum OPN concentrations in ICU patients are correlated with previously reported experimental biomarkers of critical illness and sepsis as indicated. Spearman rank correlation test, correlation coefficient r, and *p* values are given. *ICU* intensive care unit, *OPN* osteopontin

To determine factors possibly promoting elevated OPN concentrations in critically ill patients, correlation analyses with extensive sets of laboratory parameters were performed. These analyses revealed that both OPN levels at admission as well as after 3 days of ICU treatment were significantly associated with laboratory markers routinely used in the assessment of organ dysfunction. High OPN levels correlated to decreased renal function assessed by the GFR of cystatin C, elevated creatinine and urea serum concentrations (Fig. 3; Table 3). In addition to renal dysfunction, increased OPN levels at day 3 were also significantly correlated with decreased markers of liver synthesis capacity, such as albumin, pseudocholinesterase (PCHE) activity and international normalized ratio (INR). We also detected an association to indicators of cholestasis, namely bilirubin, gamma-glutamyl-transpeptidase and alkaline phosphatase (Table 3). Furthermore, serum OPN concentrations were closely correlated to markers of systemic inflammation in critically ill and sepsis patients, such as CRP, PCT, IL-6 and TNF (Table 3). Moreover, we observed a strong and significant correlation between OPN serum levels at day 3 and markers for dysregulated vital signs such as ventilation settings, serum pH and lactate levels. Consequently, we found a strong association of OPN serum concentrations at ICU admission and after 3 days of ICU treatment with established clinical scores like APACHE II, Sequential Organ Failure Assessment (SOFA) and Simplified Acute Physiology Score (SAPS2) (Fig. 3; Table 3). This important finding was further substantiated by significant correlations between OPN levels and experimental markers for organ failure and prognosis at the ICU such as APRIL, miRNA-133a, suPAR and NTproCNP, while no significant correlations with ghrelin, resistin and adiponectin were found (Fig. 3; Table 3).

**Table 3:** Correlations of osteopontin serum concentrations with other laboratory markers

	Osteopontin at admission vs. laboratory markers at admission day		Osteopontin at day 3 vs. laboratory markers at admission day		Osteopontin at day 3 vs. laboratory markers at day 3	
	r	*p*	r	*p*	r	*p*
Markers of inflammation						
CRP	0.306	0.001	0.202	0.033	0.050	0.601
Procalcitonin	0.378	0.006	0.295	0.010	0.620	<0.001
IL-10	−0.066	0.761	0.300	0.040	NA	
IL-6	−0.131	0.629	0.081	0.612	0.153	0.464
Markers of organ function						
Creatinine	0.258	0.006	0.307	0.001	0.363	<0.001
Cystatin	0.248	0.266	0.286	0.036	0.389	0.019
GFR	−0.235	0.047	−0.350	0.002	−0.390	0.001
AST	0.116	0.268	0.318	0.001	0.275	0.009
ALT	0.042	0.662	0.307	0.001	0.226	0.017
GLDH	−0.031	0.773	0.247	0.018	0.342	0.003
Bilirubin total	0.120	0.210	0.210	0.027	0.279	0.003
GGT	0.140	0.144	0.301	0.001	0.284	0.003
PCHE	−0.294	0.005	−0.159	0.126	−0.149	0.185
Albumin	−0.326	0.019	−0 206	0.090	−0.358	0.002
Urea	0.315	0.001	0.317	0.001	0.272	0.004
Iono lactate	0.040	0.696	0.349	<0.001	0.385	<0.001
LDH	0.210	0.027	0.379	<0.001	0.333	<0.001
BNP	0.224	0.045	NA		NA	
Fibrinogen	0.323	0.191	−0.269	0.057	−0.461	0.005
Clinical scoring						
APACHE II	0.292	0.012	0.230	0.036	0.294	0.062
SAPS2	0.800	0.104	0.387	0.008	0.312	0.031
SOFA	0.387	0.003	0.486	0.003	0.512	0.002
Experimental sepsis markers						
APRIL	0.258	0.040	0.325	0.005	NA	
miR-133	0.293	0.024	0.404	0.001	NA	
NTproCNP	0.284	0.003	0.325	0.001	0.395	<0.001
suPAR	0.331	0.001	0.396	<0.001	0.393	<0.001
Leptin	−0.343	0.101	−0.015	0.918	NA	
Leptin receptor	−0.249	0.241	0.260	0.075	NA	
Adiponectin	0.083	0.700	0.305	0.035	NA	
Ghrelin	0.058	0.786	−0.109	0.460	NA	
Resistin	0.205	0.336	0.226	0.122	NA	
Other						
C-peptide	0.557	0.009	0.107	0.439	0.316	0.061
Iono BZ	−0.181	0.078	0.200	0.045	0.139	0.166
Survival time	−0.002	0.989	−0.342	0.017	−0.342	0.017
Protein	−0.350	0.003	−0.344	0.001	−0.278	0.059
Ventilation time total	0.151	0.141	0.212	0.032	0.212	0.032

*NA* not assessed, ALT alanine aminotransferase, APACHE Acute Physiology and Chronic Health Evaluation, APRIL, a proliferation-inducing ligand, *AST* aspartate aminotransferase, *BNP* brain natriuretic peptide, *CRP* C-reactive protein, *GFR* glomerular filtration rate, *GGT* gamma-glutamyl transpeptidase, *GLDH* glutamate dehydrogenase, *IL* interleukin, *LDH* lactate dehydrogenase, *PCHE* pseudocholinesterase, *PCT* procalcitonin, *SAPS* Simplified Acute Physiology Score, *SOFA* Sequential Organ Failure Assessment, *SuPAR* soluble urokinase-type plasminogen activator receptor

Recent studies revealed a strong association between OPN levels and cardiac failure [14, 17]. In our cohort we were able to confirm an association between brain natriuretic peptide (BNP) and OPN serum levels at admission to the ICU. Unfortunately, in our cohort of patients, BNP concentrations were only analysed in 66 patients.

### Elevated osteopontin concentrations are associated with ICU and overall survival

**Fig. 4:**
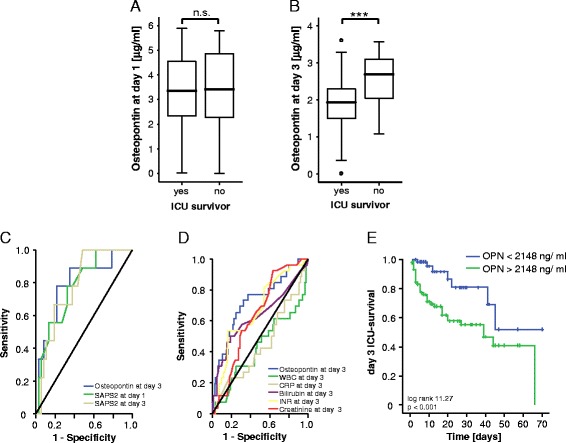
Prediction of ICU mortality by osteopontin serum concentrations. **a** OPN serum levels at admission were unchanged in patients that died during the course of ICU treatment compared to those that survived. **b** In contrast, OPN serum levels after 3 days of ICU treatment were significantly higher in patients that died during the course of ICU treatment compared to those that survived (*p* <0.001, *U* test). Box plot are displayed, where the *bold line* indicates the median per group, the *box* represents 50 % of the values, and *horizontal lines* show minimum and maximum values of the calculated non-outlier values. **c** ROC) curve analyses comparing the prognostic value of OPN levels at day 3 of ICU treatment for ICU survival (AUC 0.793) with that of the initial SAPS2 – score (AUC 0.776) and day 3 SAPS2 –score (AUC 0.781). **d** ROC curve analyses comparing the prognostic value of OPN levels at day 3 of ICU treatment for ICU survival (AUC 0.725) with that of WBC (AUC 0.431), CRP (AUC 0.447), bilirubin (AUC 0.631), INR (AUC 0.667) and creatinine (AUC 0.632). **e** Kaplan-Meier survival curves of ICU revealed that patients with OPN levels below 2148 ng/ml had a decreased ICU mortality as compared to patients with higher OPN serum concentrations. *p* values are given in the figure. ^***^ *p* <0.001. *AUC* area under the curve, *CRP* C-reactive protein, *ICU* intensive care unit, *INR* international normalized ratio, *OPN* osteopontin, *ROC* receiver operating characteristic, *SAPS* Simplified Acute Physiology Score, *WBC* white blood cell count

Based on the clear associations of serum OPN with inflammatory markers, organ dysfunction and prognostic clinical scores, we hypothesized that OPN measurements could predict the mortality risk in critically ill medical patients. We therefore first compared OPN serum concentrations at admission as well as after 3 days of ICU treatment in patients that died during the ICU treatment to those from survivors. Interestingly, patients that died on ICU displayed higher serum OPN concentrations at day 3, but not at admission, compared to survivors (Fig. 4a, b). High OPN levels at day 3 were a strong prognostic predictor for mortality at the ICU and showed a comparable prognostic accuracy like established multifactorial scores such as SAPS2 score (Fig. 4c) and APACHE II (not shown). We extended our ROC curve analysis and compared the prognostic value of OPN measurements for ICU mortality with that of serum levels of CRP and PCT representing established markers for systemic inflammation or creatinine, bilirubin and INR representing established markers for organ failure, respectively. This analysis revealed a superior performance of OPN measurements for prediction of patients prognosis compared to all of these single markers (Fig. 4d).

We next applied the approach of Ray et al. [31] to determine an optimal threshold with the highest Youden Index for OPN levels predicting the patients’ mortality during ICU treatment. This analysis revealed that an OPN concentration of 2148 ng/ml displayed the best sensitivity and specificity to predict prognosis at the ICU. Furthermore, we calculated the likelihood ratios and diagnostic odds ratio for this cutoff (Table S1 in Additional file 1) supporting our hypothesis that OPN measurements might be suitable for predicting patients’ prognosis. Based on this value we performed Kaplan-Meier survival analysis, showing significantly improved ICU survival of critically ill patients with OPN levels <2148 ng/ml at day 3 compared to patients with higher OPN concentrations at day 3 (Fig. 4e). Of note, when Kaplan-Meier curves were performed with different cutoffs such as OPN levels of the upper quartile of measurements or the clinically more convenient threshold of 2500 ng/ml, high OPN levels were consistently associated with an impaired prognosis (Figure S1 in Additional file 2). In line, survivors displayed a tendency towards a stronger decrease in OPN serum concentrations from admission to day 3 compared to non-survivors (not shown). Based on post hoc analyses, we finally attempted to evaluate a “grey zone” with two additional cutoffs to detect ICU mortality. An OPN level of 2786 ng/ml is the cutoff level corresponding to a high specificity (sensitivity: 0.38; specificity 0.85), while 1653 ng/ml is the OPN cutoff value corresponding to a high sensitivity (sensitivity: 0.85; specificity 0.36; Table S1 in Additional file 1).

To further substantiate these results on a potential prognostic value of OPN measurements we next performed a multivariate Cox regression analysis including markers of inflammation/infection (CRP, white blood cell count (WBC)), hepatic (bilirubin, INR) and renal (creatinine) function at day 3, OPN was identified as an independent significant prognostic parameter to predict ICU survival (Table 4).

**Table 4:** Multivariate Cox regression analysis for osteopontin levels at admission to predict ICU survival

Parameter	Unadjusted HR (95 % CI)	*p* value	Adjusted HR (95 % CI)	*p* value
CRP	-	n.s.	-	n.s.
WBC	-	n.s.	-	n.s.
Bilirubin	1.079 (1.039–1.121)	<0.001	-	n.s.
INR	2.041 (1.435–2.902)	<0.001	1.782 (1.044–3.041)	0.034
Creatinine	-	n.s.	-	n.s.
Osteopontin	1.001 (1.000–1.001)	0.009	1.001 (1.000–1.001)	0.009

*CI* confidence interval, *CRP* C-reactive protein, *INR* international normalized ratio, *HR* hazard ratio, *WBC* white blood cell count

**Fig. 5:**
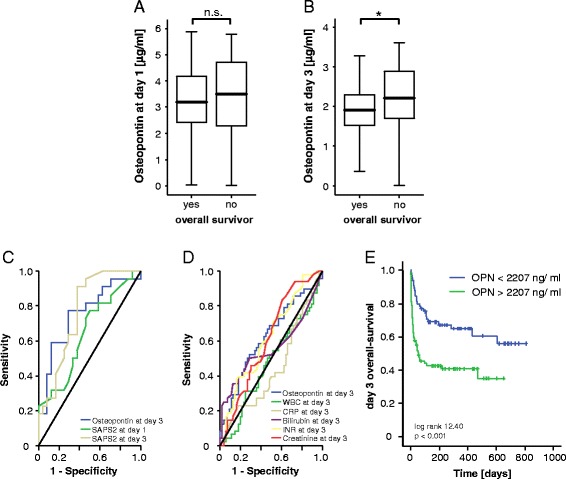
Prediction of long-term mortality by osteopontin serum concentrations. **a** and **b** High serum OPN serum levels indicate an impaired outcome in critically ill patients as patients that survived ICU treatment had lower OPN serum levels after 3 days of ICU treatment (*p* = 0.035, *U* test). Box plot are displayed, where the *bold line* indicates the median per group, the *box* represents 50 % of the values, and *horizontal lines* show minimum and maximum values of the calculated non-outlier values. **c** ROC curve analyses comparing the prognostic value of OPN levels at day 3 of ICU treatment for overall survival (AUC 0.752) with that of the initial SAPS2 – score (AUC 0.647) and day 3 SAPS2 – score (AUC 0.768). **d** ROC curve analyses comparing the prognostic value of OPN levels at day 3 of ICU treatment for overall survival (AUC 0.626) with that of WBC (AUC 0.475), CRP (AUC 0.446), bilirubin (AUC 0.560), INR (AUC 0.621) and creatinine (AUC 0.611). **e** Kaplan-Meier survival curves of ICU patients showed that patients with high OPN concentrations had an increased overall mortality compared to other patients. *p* values for log rank test are given in the figure. ^*^ *p* <0.05. *AUC* area under the curve, *CRP* C-reactive protein, *ICU* intensive care unit, *INR* international normalized ratio, *OPN* osteopontin, *ROC* receiver operating characteristic, *SAPS* Simplified Acute Physiology Score, *WBC* white blood cell count

In our cohort of critically ill patients, 25.8 % died at the ICU, while the overall mortality rate increased to 46.5 % during the (post-ICU/post-hospital) observation period. Patients that died during long-term follow-up showed a trend towards higher initial OPN levels than survivors; strikingly, serum OPN levels at day 3 were significantly higher levels in patients that died during follow-up than for surviving patients (Fig. 5a, b). ROC curve analysis revealed a rather low prognostic power of OPN for predicting long-term mortality (Fig. 5c), which was still superior to that of CRP and PCT as established markers of SIRS/sepsis or other markers of organ failure (Fig. 5d). Considering that high OPN was associated with overall mortality by Cox regression analysis, we calculated again an optimal threshold for OPN concentrations predicting patients’ long-term survival, revealing that an OPN concentration of 2207 ng/ml displayed the best sensitivity and specificity to predict the patients’ long-term prognosis. Kaplan-Meier curve analysis for this threshold, for the threshold of 2500 ng/ml or OPN values of the upper quartile of all measurements indicated that patients with low OPN levels had a more favorable prognosis than those with higher values (Fig. 5e, Figure S1 in Additional file 2). Patients with OPN levels >2207 ng/ml showed a significantly higher mortality rate further underscoring the value of OPN measurements at day 3 of ICU treatment for the prediction of patients’ prognosis (57 versus 46 %). Youden Index as well as the likelihood ratios and diagnostic odds ratio for this cutoff are given in Table S2 in Additional file 1. Again, we evaluated a “grey zone” with two additional cutoffs to detect overall death. The first cutoff with OPN level of 2808 ng/ml corresponds to a high specificity (sensitivity: 0.29; specificity 0.86). The second cutoff 1486 ng/ml corresponds to a high sensitivity (sensitivity: 0.86; specificity 0.21; Table S1 in Additional file 1).

Of note, multivariate Cox regression analyses, including markers of inflammation/infection (CRP, WBC), hepatic (bilirubin, INR) and renal (creatinine) function at day 3, revealed that OPN represents an independent significant prognostic parameter also for overall survival (Table 5).

**Table 5:** Multivariate Cox regression analysis for osteopontin levels at admission to predict overall survival

Parameter	Unadjusted HR (95 % CI)	*p* value	Adjusted HR (95 % CI)	*p* value
CRP	-	n.s.	-	n.s.
WBC	-	n.s.	-	n.s.
Bilirubin	-	n.s.	1.056 (1.006–1.109)	0.028
INR	-	n.s.	-	n.s.
Creatinine	-	n.s.	-	n.s.
Osteoponin	1.001 (1.000–1.001)	0.004	1.001 (1.000–1.001)	0.014

*CI* confidence interval, *CRP* C-reactive protein, *INR* international normalized ratio, *HR* hazard ratio, *WBC* white blood cell count

Thus, our data indicate that measurement of OPN levels in a medical ICU environment might have a valuable role in the assessment of a critically ill patient’s short-term and long-term prognosis.

## Discussion

In this study, we assessed OPN concentrations upon admission to the medical ICU before specific therapeutic interventions and at day 3 after ICU admission in a well-characterized cohort of critically ill patients [23, 25, 33, 34]. In these patients, OPN serum concentrations were found to have a close association with the prognosis, especially if assessed at day 3 after admission to the ICU. OPN is a secreted phosphorylated protein that exists as a component of the extracellular matrix and as a soluble cytokine. Under basal conditions, OPN is biosynthesized by various tissue types including osteocytes, fibroblasts, osteoblasts, smooth muscle and endothelial cells [35–38]. In the immune system, it is expressed by many cell types like macrophages, neutrophils, dendritic cells, natural killer (NK) cells and T and B lymphocytes [39]. OPN’s capacity to interact with multiple surface receptors suggests that it is an active player in many physiological and pathological processes. It is upregulated due to many different stress stimuli, implying a functional role during stress response [39]. Its functional relevance in immunity and during the inflammatory response to infection and cell damage are well documented [39, 40]. For instance, OPN plays an important role in chemotaxis and recruitment of neutrophils and macrophages. Moreover, it modulates cell-mediated immune reactions by promoting the response of T helper (Th1)-type CD4^+^ T cells and driving IL-17 expression [41]. Importantly, OPN modulates immunity in different directions. Although it is widely designated as a pro-inflammatory factor, it can also mediate anti-inflammatory effects under certain conditions [39].

Sepsis and the Systemic Inflammatory Response Syndrome (SIRS) represent states of profound dysbalance of the immune system in response to infection and/or organ damage, menacing the prognosis of many patients referred to the ICU [23]. The exact pathomechanisms of sepsis/SIRS are not yet fully understood. The clinical picture is determined by an excessive inflammatory response of the immune system to the triggering stimulus, followed by a prolonged immunosuppressive state [42]. Both stages of the disease process seem to comprise the prognosis. However, recent reports imply that especially the effects of the delayed immunosuppressive phase may have been underestimated [42]. Both the initiation and progression of sepsis seems to be orchestrated by activated T cells, particularly CD4^+^ T helper 1 (Th1) and Th2 cells [43]. Moreover, Th17 cells that produce IL-17 have been demonstrated to play an important role in the regulation of pro- and anti-inflammatory factors during sepsis [43]. OPN, that is also known as Eta-1 (early T lymphocyte activation gene 1), is highly expressed in activated T cells. It has been shown to regulate CD4+ T helper cell lineage commitment towards the specific Th subtypes [9] and to drive IL-17 production [41]. Thus, it seems obvious that OPN exerts important regulatory functions in the pathogenesis of sepsis. In this regard, Vaschetto and co-workers have demonstrated increased serum levels of OPN in patients suffering from sepsis and SIRS compared to healthy controls [18]. On the one hand, our data confirm this report showing elevated OPN levels compared to healthy controls and lower levels of OPN in surviving versus non-surviving patients. Moreover, our work extends the results from Vaschetto et al., demonstrating that OPN correlated significantly better with the prognosis than “classical” prognostic markers like CRP, INR and creatinine and also than the sepsis marker PCT.

Of note, Vaschetto et al. even suggested that OPN serum levels may allow differentiating between sepsis and SIRS [18]. In contrast, we could not recapitulate a strong specificity of OPN for the diagnosis of sepsis. In our study comprising a heterogeneous medical population of critically ill patients, there was no significant difference of OPN levels between patients with or without sepsis. Our results indeed indicate a fundamental role of OPN in the pathogenesis of inflammatory dysbalance of ICU patients, independent of the presence of an infection. Thus, our report might support the recently growing notion that the course and prognosis of disease patterns of ICU patients, like cardiogenic shock or liver failure, seem to be mainly determined by the patient’s inflammatory response that self-dynamically develops beyond the initial pathogenic stimulus [44–46]. In line with this notion, OPN serum levels correlated both with markers of liver failure (alkaline phosphatase (AP); gamma glutamyl transpeptidase (GGT), bilirubin) heart failure (BNP), clinical scores like APACHE II and SAPS2 as well as novel prognostic markers like APRIL [23] and circulating miR-133a [25]. In this regard, it is not surprising that OPN is highly upregulated in conditions of critical illness upon admission to the ICU. The striking fact that patients with persistently elevated OPN levels (at day 3 of ICU treatment), in which OPN do not regress as usually seen in the cohort (Fig. 2), have a poor prognosis, is certainly very interesting to investigate on possible detrimental functions of persistently elevated OPN during systemic inflammation.

In spite of advances in diagnosis and treatment of critically ill patients throughout the recent decades, the triage, diagnostic and therapeutic management of these patients during the first week of ICU treatment still represents a major challenge. The promptness and accuracy of the initial decisions during the initial course of disease are of immense importance for the subsequent outcome of sepsis [47] or cardiogenic shock [48]. Inversely, failure of initiating the adequate therapy during the first phase of the disease may critically affect the mortality of these patients [23]. In this respect, the use of novel biomarkers that allow rapid decision-making with sufficient accuracy may significantly improve the treatment and finally the outcome of ICU patients [49, 50]. OPN serum levels seem to specifically unfold its power to predict the prognosis of patients in the early phase after ICU admission, thus offering a novel tool to guide treatment decisions at this critical time-point. Given that OPN at day 3 of ICU treatment is a strong predictor of mortality risk, one could speculate that its use might be implemented into established scoring systems together with markers that detect the initial cause of the critical illness leading to ICU admission (e.g. APRIL, which has recently been demonstrated to specifically detect sepsis [23], or BNP as a marker of heart failure [51]).

Taken together, our data provide evidence for a role of OPN as a diagnostic tool in the prognostic judgment of critically ill and septic patients during the early phase of their ICU stay. Certainly, these data need to be recapitulated in larger independent cohorts as well as in other ICU settings such as post-surgery care, before implementation into clinical algorithms can be considered. Moreover, although our data imply an important role of OPN in the molecular pathogenesis in critically ill patients, they do not provide a specific molecular mechanism of action. OPN may trigger either pro-inflammatory stimuli like TNF or IL-6 (Table 3) as well as anti-inflammatory stimuli [9]. In this regard, a recent study by Fortis et al. demonstrated that OPN is required for enhanced glucocorticosteroid production in an animal model of sepsis [52]. In line with the results from our report, the authors demonstrate that OPN is associated with a worsened outcome in sepsis in spite of enhanced glucocorticoid levels, which are thought to have a beneficial impact in severe sepsis [52]. One might speculate that in the setting of sepsis, potentially beneficial targets of OPN are outbalanced by maladaptive effects. In our study, we assessed a more heterogeneous population of critically ill patients with different entities, including sepsis, but also cardiogenic shock or liver failure. The balance between pro- and anti-inflammatory effects of OPN might be specifically regulated in each of these entities. Nevertheless, the results from our study provide a clear rationale for future functional studies on the role of OPN in different disease models related to critical illness.

## Conclusions

Our study identifies osteopontin (OPN) as a stable and robust marker in critically ill patients to assess disease severity and mortality risks. While OPN serum levels are elevated compared to healthy controls both at admission to the ICU and after 3 days of treatment, persistently elevated OPN levels at day 3 of ICU treatment are a strong independent predictor for an unfavourable prognosis. In line with prognostic properties, OPN is closely correlated to established prognosis scores. High OPN levels do not discriminate septic patients from non- septic patients. In contrast, OPN correlates with experimental markers of general inflammation and multi-organ failure.

## Key messages

- Serum levels of OPN in critically ill patients are elevated compared to healthy controls both at admission to the ICU and after 3 days of treatment.
- OPN concentrations were related to disease severity and significantly correlated with established prognosis scores and classical as well as experimental markers of inflammation and multi-organ failure.
- In the total cohort, OPN levels decreased from admission to day 3 of ICU treatment. However, persistently elevated OPN levels at day 3 of ICU treatment were a strong independent predictor for an unfavourable prognosis and indicated short- and long-term mortality with higher diagnostic accuracy than routinely used markers of organ failure and prognostic scoring systems such as SAPS2 or APACHE II score.

## Additional files

Additional file 1: Table S1. Cutoff values for OPN at day 3 to distinguish between ICU survivors yes vs. no; **Table S2.** Cutoff values for OPN at day 3 to distinguish between overall survivors yes vs. no. Additional file 2: Figure S1. Elevated osteopontin serum levels indicate poor survival in critically ill patients. **a** Kaplan-Meier survival curves of ICU patients showing that patients with OPN levels within the lower quartile of all patients had a decreased short-term mortality at the ICU as compared to other patients. **b** Kaplan-Meier survival curves of ICU revealed that patients with OPN levels below 2500 ng/ml had a decreased ICU mortality as compared to patients with higher OPN serum concentrations. **c** Kaplan-Meier curve analysis of ICU patients demonstrates that patients with OPN levels within the upper quartile had a decreased overall survival as compared to other patients. **d** Kaplan-Meier survival curves of ICU patients showed that patients with high OPN concentrations had an increased overall mortality compared to other patients. *p* values are given in the figure.

## Abbreviations

ALT: alanine aminotransferase
APACHE: Acute Physiology and Chronic Health Evaluation
APRIL: a proliferation-inducing ligand
AST: aspartate aminotransferase
AUC: area under the curve
BMI: body mass index
BNP: brain natriuretic peptide
CRP: C-reactive protein
ELISA: enzyme-linked immunosorbent assay
GFR: glomerular filtration rate
GGT: gamma glutamyl transpeptidase
GLDH: glutamate dehydrogenase
ICU: intensive care unit
IL: interleukin
INR: international normalized ratio
LDH: lactate dehydrogenase
LHR-: negative likelihood ratio
LHR+: positive likelihood ratio
miRNA: micro ribonucleic acid
NK: natural killer
OPN: osteopontin
PCHE: pseudocholinesterase
PCT: procalcitonin
RNA: ribonucleic acid
ROC: receiver operating characteristic
SAPS: Simplified Acute Physiology Score
SIRS: Systemic Inflammatory Response Syndrome
SOFA: Sequential Organ Failure Assessment
SuPAR: soluble urokinase-type plasminogen activator receptor
Th: T helper
TNF: tumour necrosis factor
WBC: white blood cell count

## References

[1] Stevenson EK, Rubenstein AR, Radin GT, Wiener RS, Walkey AJ (2014). Two decades of mortality trends among patients with severe sepsis: A comparative meta-analysis*. Crit Care Med.

[2] Bone RC, Balk RA, Cerra FB, Dellinger RP, Fein AM, Knaus WA (1992). Definitions for sepsis and organ failure and guidelines for the use of innovative therapies in sepsis. The ACCP/SCCM Consensus Conference Committee. Am Coll Chest Phys.

[3] Hotchkiss RS, Monneret G, Payen D (2013). Sepsis-induced immunosuppression: from cellular dysfunctions to immunotherapy. Nat Rev Immunol.

[4] Liu X, Ren H, Peng D (2014). Sepsis biomarkers: an omics perspective. Front Med.

[5] Bandopadhyay M, Bulbule A, Butti R, Chakraborty G, Ghorpade P, Ghosh P (2014). Osteopontin as a therapeutic target for cancer. Expert Opin Ther Targets.

[6] Morimoto J, Kon S, Matsui Y, Uede T (2010). Osteopontin; as a target molecule for the treatment of inflammatory diseases. Curr Drug Targets.

[7] Singh M, Foster CR, Dalal S, Singh K (2010). Osteopontin: role in extracellular matrix deposition and myocardial remodeling post-MI. J Mol Cell Cardiol.

[8] Scatena M, Liaw L, Giachelli CM (2007). Osteopontin: a multifunctional molecule regulating chronic inflammation and vascular disease. Arterioscler Thromb Vasc Biol.

[9] Lund SA, Giachelli CM, Scatena M (2009). The role of osteopontin in inflammatory processes. J Cell Commun Sign.

[10] Koguchi Y, Kawakami K, Uezu K, Fukushima K, Kon S, Maeda M (2003). High plasma osteopontin level and its relationship with interleukin-12-mediated type 1 T helper cell response in tuberculosis. Am J Respir Crit Care Med.

[11] Shimizu Y, Ota K, Ikeguchi R, Kubo S, Kabasawa C, Uchiyama S (2013). Plasma osteopontin levels are associated with disease activity in the patients with multiple sclerosis and neuromyelitis optica. J Neuroimmunol.

[12] Wong CK, Lit LC, Tam LS, Li EK, Lam CW (2005). Elevation of plasma osteopontin concentration is correlated with disease activity in patients with systemic lupus erythematosus. Rheumatology.

[13] Mishima R, Takeshima F, Sawai T, Ohba K, Ohnita K, Isomoto H (2007). High plasma osteopontin levels in patients with inflammatory bowel disease. J Clin Gastroenterol.

[14] Behnes M, Brueckmann M, Lang S, Espeter F, Weiss C, Neumaier M (2013). Diagnostic and prognostic value of osteopontin in patients with acute congestive heart failure. Eur J Heart Fail.

[15] Rosenberg M, Meyer FJ, Gruenig E, Lutz M, Lossnitzer D, Wipplinger R (2012). Osteopontin predicts adverse right ventricular remodelling and dysfunction in pulmonary hypertension. Eur J Clin Invest.

[16] Rosenberg M, Meyer FJ, Gruenig E, Schuster T, Lutz M, Lossnitzer D (2012). Osteopontin (OPN) improves risk stratification in pulmonary hypertension (PH). Int J Cardiol.

[17] Rosenberg M, Zugck C, Nelles M, Juenger C, Frank D, Remppis A (2008). Osteopontin, a new prognostic biomarker in patients with chronic heart failure. Circ Heart Fail.

[18] Vaschetto R, Nicola S, Olivieri C, Boggio E, Piccolella F, Mesturini R (2008). Serum levels of osteopontin are increased in SIRS and sepsis. Intensive Care Med.

[19] Gressner OA, Koch A, Sanson E, Trautwein C, Tacke F (2008). High C5a levels are associated with increased mortality in sepsis patients–no enhancing effect by actin-free Gc-globulin. Clin Biochem.

[20] Koch A, Voigt S, Kruschinski C, Sanson E, Duckers H, Horn A (2011). Circulating soluble urokinase plasminogen activator receptor is stably elevated during the first week of treatment in the intensive care unit and predicts mortality in critically ill patients. Crit Care.

[21] Dellinger RP, Levy MM, Carlet JM, Bion J, Parker MM, Jaeschke R (2008). Surviving Sepsis Campaign: international guidelines for management of severe sepsis and septic shock: 2008. Crit Care Med.

[22] Reinhart K, Brunkhorst FM, Bone HG, Bardutzky J, Dempfle CE, Forst H, et al. Prevention, diagnosis, therapy and follow-up care of sepsis: 1st revision of S-2k guidelines of the German Sepsis Society (Deutsche Sepsis-Gesellschaft e.V. (dsg)) and the German Interdisciplinary Association of Intensive Care and Emergency Medicine (Deutsche Interdisziplinare Vereinigung fur Intensiv - und Notfallmedizin (DIVI)). Ger Med Sci. 2010;8:Doc14. 10.3205/000103 PMC2899863 20628653

[23] Roderburg C, Koch A, Tacke F, Nieuwenhuijsen L, Bruensing J, Vargas Cardenas D (2013). Serum concentrations of a proliferation-inducing ligand (April) are elevated in sepsis and predict mortality in critically ill patients. J Crit Care.

[24] Benz F, Roderburg C, Vargas Cardenas D, Vucur M, Gautheron J, Koch A (2013). U6 is unsuitable for normalization of serum miRNA levels in patients with sepsis or liver fibrosis. Exp Mol Med.

[25] Tacke F, Roderburg C, Benz F, Cardenas DV, Luedde M, Hippe HJ (2014). Levels of circulating miR-133a are elevated in sepsis and predict mortality in critically ill patients. Crit Care Med.

[26] Koch A, Voigt S, Sanson E, Duckers H, Horn A, Zimmermann HW (2011). Prognostic value of circulating amino-terminal pro-c-type natriuretic peptide in critically ill patients. Crit Care.

[27] Koch A, Sanson E, Helm A, Voigt S, Trautwein C, Tacke F (2010). Regulation and prognostic relevance of serum ghrelin concentrations in critical illness and sepsis. Crit Care.

[28] Koch A, Zimmermann HW, Baeck C, Schneider C, Yagmur E, Trautwein C (2012). Serum NT-proCNP concentrations are elevated in patients with chronic liver diseases and associated with complications and unfavorable prognosis of cirrhosis. Clin Biochem.

[29] Yagmur E, Koch A, Haumann M, Kramann R, Trautwein C, Tacke F (2012). Hyaluronan serum concentrations are elevated in critically ill patients and associated with disease severity. Clin Biochem.

[30] Roderburg C, Benz F, Vargas Cardenas D, Koch A, Janssen J, Vucur M (2015). Elevated miR-122 serum levels are an independent marker of liver injury in inflammatory diseases. Liver Int.

[31] Ray P, Le Manach Y, Riou B, Houle TT (2010). Statistical evaluation of a biomarker. Anesthesiology.

[32] Gordin D, Forsblom C, Panduru NM, Thomas MC, Bjerre M, Soro-Paavonen A (2014). Osteopontin is a strong predictor of incipient diabetic nephropathy, cardiovascular disease, and all-cause mortality in patients with type 1 diabetes. Diabetes Care.

[33] Koch A, Weiskirchen R, Bruensing J, Duckers H, Buendgens L, Kunze J (2013). Regulation and prognostic relevance of symmetric dimethylarginine serum concentrations in critical illness and sepsis. Mediators Inflamm.

[34] Roderburg C, Luedde M, Vargas Cardenas D, Vucur M, Scholten D, Frey N (2013). Circulating microRNA-150 serum levels predict survival in patients with critical illness and sepsis. PLoS One.

[35] Ashizawa N, Graf K, Do YS, Nunohiro T, Giachelli CM, Meehan WP (1996). Osteopontin is produced by rat cardiac fibroblasts and mediates A(II)-induced DNA synthesis and collagen gel contraction. J Clin Invest.

[36] Ikeda T, Shirasawa T, Esaki Y, Yoshiki S, Hirokawa K (1993). Osteopontin mRNA is expressed by smooth muscle-derived foam cells in human atherosclerotic lesions of the aorta. J Clin Invest.

[37] Murry CE, Giachelli CM, Schwartz SM, Vracko R (1994). Macrophages express osteopontin during repair of myocardial necrosis. Am J Pathol.

[38] Uaesoontrachoon K, Yoo H-J, Tudor EM, Pike RN, Mackie EJ, Pagel CN (2008). Osteopontin and skeletal muscle myoblasts: association with muscle regeneration and regulation of myoblast function in vitro. Int J Biochem Cell Biol.

[39] Wang KX, Denhardt DT (2008). Osteopontin: role in immune regulation and stress responses. Cytokine Growth Factor Rev.

[40] Denhardt DT, Giachelli CM, Rittling SR (2001). Role of osteopontin in cellular signaling and toxicant injury. Annu Rev Pharmacol Toxicol.

[41] Steinman L (2007). A brief history of T(H)17, the first major revision in the T(H)1/T(H)2 hypothesis of T cell-mediated tissue damage. Nat Med.

[42] Remick DG (2003). Cytokine therapeutics for the treatment of sepsis: why has nothing worked?. Curr Pharm Des.

[43] Rittirsch D, Flierl MA, Ward PA (2008). Harmful molecular mechanisms in sepsis. Nat Rev Immunol.

[44] Reynolds HR, Hochman JS (2008). Cardiogenic shock: current concepts and improving outcomes. Circulation.

[45] Shawcross DL, Sharifi Y, Canavan JB, Yeoman AD, Abeles RD, Taylor NJ (2011). Infection and systemic inflammation, not ammonia, are associated with Grade 3/4 hepatic encephalopathy, but not mortality in cirrhosis. J Hepatol.

[46] Shpektor A (2010). Cardiogenic shock: the role of inflammation. Acute Card Care.

[47] Gaieski DF, Mikkelsen ME, Band RA, Pines JM, Massone R, Furia FF (2010). Impact of time to antibiotics on survival in patients with severe sepsis or septic shock in whom early goal-directed therapy was initiated in the emergency department. Crit Care Med.

[48] Harker M, Carville S, Henderson R, Gray H, Guideline DG (2014). Key recommendations and evidence from the NICE guideline for the acute management of ST-segment-elevation myocardial infarction. Heart.

[49] Balmelli C, Drexler B, Mueller C (2011). Utile or futile: Biomarkers in the ICU. Crit Care.

[50] Noveanu M, Mebazaa A, Mueller C (2009). Cardiovascular biomarkers in the ICU. Curr Opin Crit Care.

[51] Maisel AS, Choudhary R (2012). Biomarkers in acute heart failure–state of the art. Nat Rev Cardiol.

[52] Fortis S, Khadaroo RG, Haitsma JJ, Zhang H (2015). Osteopontin is associated with inflammation and mortality in a mouse model of polymicrobial sepsis. Acta Anaesthesiol Scand.

