# Supplementary material for: Persistently elevated osteopontin serum levels predict mortality in critically ill patients
Source: Crit Care. 2015 Jun 26;19(1):271. doi: 10.1186/s13054-015-0988-4 (PMC4490692; doi:10.1186/s13054-015-0988-4)
Supplement: Additional file 1: Table S1. — Cutoff values for OPN at day 3 to distinguish between ICU survivors yes vs. no; Table S2. Cutoff values for OPN at day 3 to distinguish between overall survivors yes vs. no. [file 13054_2015_988_MOESM1_ESM.pdf]

Suppl. Table 1: Cutoff values for OPN at day 3 to distinguish between ICU- survivors yes vs. no

**Cutoff value [ng/ml]**	**sensitivity**	**specificity**	**Youden index**	**LHR+**	**LHR-**	**diagnostic odds ratio**
2148	0.73	0.69	0.42	2.33	0.39	5.93
2786	0.38	0.85	0.23	2.54	0.72	3.51
1653	0.85	0.36	0.21	1.32	0.43	3.10

Suppl. Table 2: Cutoff values for OPN at day 3 to distinguish between overall- survivors yes vs. no

**Cutoff value [ng/ml]**	**sensitivity**	**specificity**	**Youden index**	**LHR+**	**LHR-**	**diagnostic odds ratio**
2208	0.51	0.72	0.23	1.82	0.68	2.67
2808	0.29	0.86	0.15	2.04	0.83	2.45
1486	0.86	0.21	0.07	1.09	0.68	1.60
