# Supplementary material for: Persistently elevated osteopontin serum levels predict mortality in critically ill patients
Source: Crit Care. 2015 Jun 26;19(1):271. doi: 10.1186/s13054-015-0988-4 (PMC4490692; doi:10.1186/s13054-015-0988-4)
Supplement: Additional file 2: Figure S1. — Elevated osteopontin serum levels indicate poor survival in critically ill patients. a Kaplan-Meier survival curves of ICU patients showing that patients with OPN levels within the lower quartile of all patients had a decreased short-term mortality at the ICU as compared to other patients. b Kaplan-Meier survival curves of ICU revealed that patients with OPN levels below 2500 ng/ml had a decreased ICU mortality as compared to patients with higher OPN serum concentrations. c Kaplan-Meier curve analysis of ICU patients demonstrates that patients with OPN levels within the upper quartile had a decreased overall survival as compared to other patients. d Kaplan-Meier survival curves of ICU patients showed that patients with high OPN concentrations had an increased overall mortality compared to other patients. p values are given in the figure. [file 13054_2015_988_MOESM2_ESM.pptx]

## Slide 1
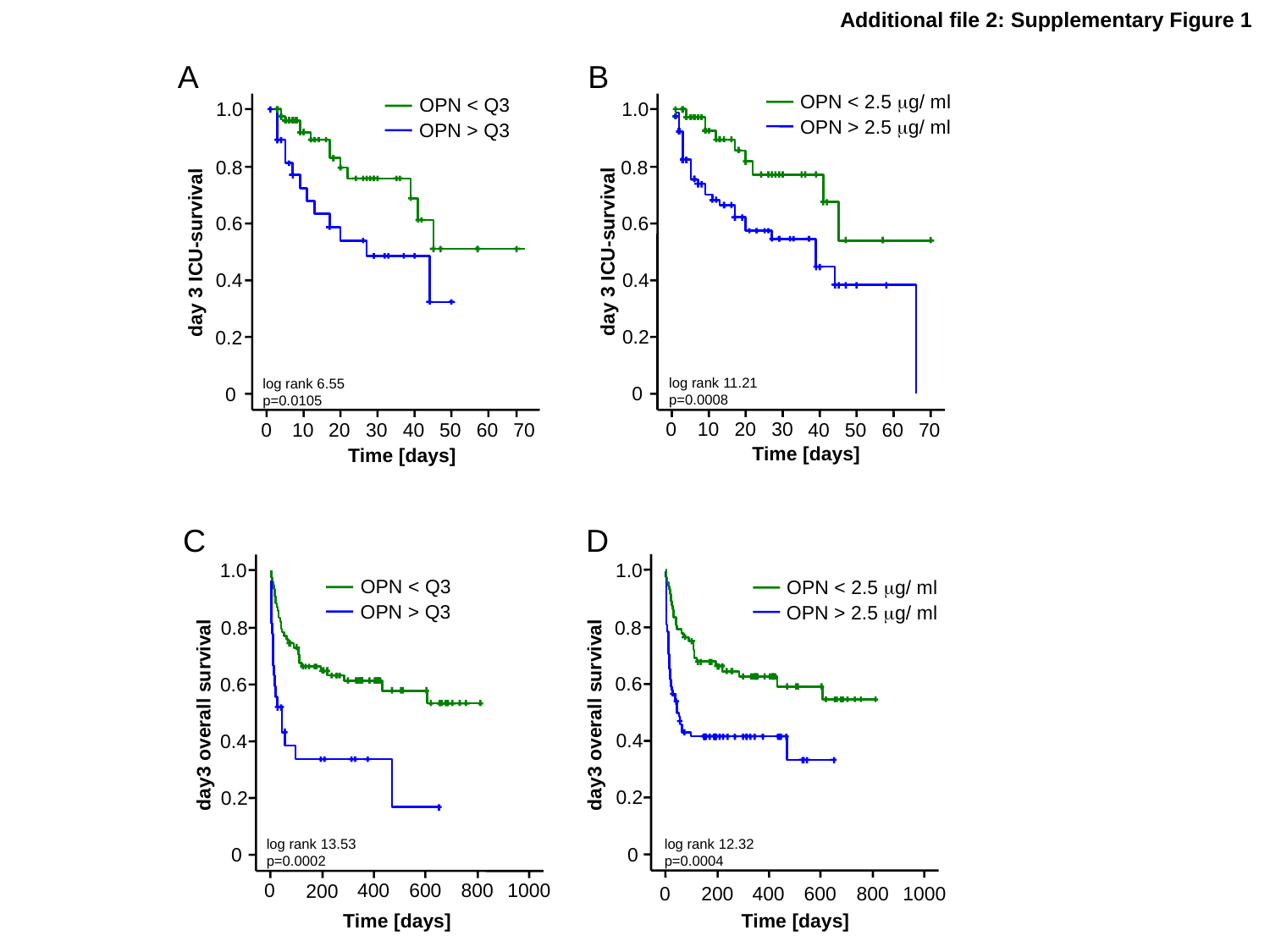

Additional file 2: Supplementary Figure 1
A
B
 OPN < 2.5 μg/ ml
 OPN < Q3
1.0
1.0
OPN > 2.5 μg/ ml
OPN > Q3
0.8
0.8
0.6
0.6
day 3 ICU-survival
day 3 ICU-survival
0.4
0.4
0.2
0.2
log rank 11.21
p=0.0008
log rank 6.55
p=0.0105
0
0
0
10
20
30
0
10
20
30
40
50
60
70
40
50
60
70
Time [days]
Time [days]
C
D
1.0
1.0
 OPN < Q3
 OPN < 2.5 μg/ ml
OPN > Q3
OPN > 2.5 μg/ ml
0.8
0.8
0.6
0.6
day3 overall survival
day3 overall survival
0.4
0.4
0.2
0.2
log rank 13.53
p=0.0002
log rank 12.32
p=0.0004
0
0
0
400
600
800
1000
200
0
200
400
600
800
1000
Time [days]
Time [days]
